# Baicalein Inhibits Progression of Gallbladder Cancer Cells by Downregulating ZFX

**DOI:** 10.1371/journal.pone.0114851

**Published:** 2015-01-24

**Authors:** Tian-Yu Liu, Wei Gong, Zhu-Jun Tan, Wei Lu, Xiang-Song Wu, Hao Weng, Qian Ding, Yi-Jun Shu, Run-Fa Bao, Yang Cao, Xu-An Wang, Fei Zhang, Huai-Feng Li, Shan-Shan Xiang, Lin Jiang, Yun-ping Hu, Jia-Sheng Mu, Mao-Lan Li, Wen-Guang Wu, Bai-Yong Shen, Li-Xin Jiang, Ying-Bin Liu

**Affiliations:** 1 Institute of Biliary Tract Disease, Shanghai Jiao Tong University School of Medicine, Shanghai, China; 2 Laboratory of General Surgery, Xinhua Hospital, Affiliated to Shanghai Jiao Tong University, School of Medicine, Shanghai, China; 3 Department of General Surgery, Xinhua Hospital, Affiliated to Shanghai Jiao Tong University, School of Medicine, No.1665 Kongjiang Road, Shanghai 200092, China; 4 Department of General Surgery, Ruijin Hospital Affiliated to Shanghai Jiao Tong University, 197 Ruijin Er Road, Shanghai 200025, China; 5 Department of General Surgery, Jiangyin hospital of traditional Chinese medicine, Jiangyin, China; Kyung Hee University, KOREA, REPUBLIC OF

## Abstract

Baicalein, a widely used Chinese herbal medicine, has multiple pharmacological activities. However, the precise mechanisms of the anti-proliferation and anti-metastatic effects of baicalein on gallbladder cancer (GBC) remain poorly understood. Therefore, the aim of this study was to assess the anti-proliferation and anti-metastatic effects of baicalein and the related mechanism(s) on GBC. In the present study, we found that treatment with baicalein induced a significant inhibitory effect on proliferation and promoted apoptosis in GBC-SD and SGC996 cells, two widely used gallbladder cancer cell lines. Additionally, treatment with baicalein inhibited the metastasis of GBC cells. Moreover, we demonstrated for the first time that baicalein inhibited GBC cell growth and metastasis via down-regulation of the expression level of Zinc finger protein X-linked (ZFX). In conclusion, our studies suggest that baicalein may be a potential phytochemical flavonoid for therapeutics of GBC and ZFX may serve as a molecular marker or predictive target for GBC.

## Introduction

Gallbladder cancer (GBC) is the fifth most common cancer of the biliary tract, characterized by early lymph node invasion and distant metastases[1–3]. It tends to be an aggressive tumor that spreads early and 90% of GBC patients are presented at an advanced, inoperable stage[4, 5]. Early gallbladder carcinoma is asymptomatic or manifests only as an abdominal discomfort. Some patients can develop the symptom of acute or chronic cholecystitis, which is easy to ignore or miss. In the later period, patients can develop abdominal pain, jaundice, and angular, but most of the patients have no surgical opportunities. The prognosis of advanced gallbladder carcinoma is very poor[6–10], and the 5-year survival rate is only about 5%. So far, surgical resection is the only treatment that offers a hope for cure[11]. Therefore it is very important to identify reliable biomarkers and drugs for monitoring both progression and treatment of the disease.

Baicalein is one of the effective ingredients extracted from *labiatae* plants *scutellaria baicalensis georgi*’s dry root, which has many pharmacological effects such as anti-inflammatory, anti-bacterial, anti-viral, anti-allergy, anti-oxidation and so on[12–14]. Recently, the anti-tumor effect of baicalein has caused more and more attention of people. Cellular and animal experiments have demonstrated that baicalein had strong anti-tumor activity. For example, baicalein activated AhR and faciliated the degradation of cyclin D1, which causes cell cycle arrest at the G1 phase, and induces the inhibition of cell proliferation[15]. Baicalein inhibits DMBA/TPA-induced skin tumorigenesisin mice by modulating proliferation, apoptosis, and inflammation[16]. Baicalein abrogates reactive oxygen species (ROS)-mediated mitochondrial dysfunction during experimental pulmonary carcinogenesis *in vivo*[17]. Baicalein may play an important role in inhibitory effect on proliferation of ovarian cancer cells[18]. Because of its wide anti-tumor spectrum, adequate medicine source, little toxicity and side effects, baicalein seems to be a promising molecular marker or therapeutic target of gallbladder cancer.

Zinc finger protein, X-linked (ZFX) is a transcription factor encoded by its gene on the mammalian X chromosome. The ZFX plays a key role in controlling the self-renew and maintenance of both embryonic stem cells and hematopoietic stem cells[19–23]. The expression level of ZFX correlates with aggressiveness and severity in many cancer types, including prostate cancer, breast cancer, human malignant glioma and leukemia. Previous research showed that ZFX may play a critical role in cell proliferation, cell cycle distribution, and apoptosis in many cancer cells[24–29]. Our previously results also found that ZFX may involve in regulation of cell proliferation and migration in gallbladder cancer[30]. Thus, the present study was designed to investigate the effects and mechanisms of baicalein against gallbladder cancer cell proliferation, invasion and metastasis *in vitro* and in an orthotopic gallbladder tumor model *in vivo*. Furthermore, the ZFX had been demonstrated as the downstream target of baicalein, which may offer a promising new approach in the effective treatment of gallbladder carcinoma.

## Materials and Methods

### Ethics statement

Nude mice (with an initial body weight of 20–22 g) were obtained from Shanghai SLAC Laboratory Animal Co., Ltd. (Shanghai, China). All animal treatments were performed strictly in accordance with international ethical guidelines and the National Institutes of Health Guide concerning the Care and Use of Laboratory Animals. The experiments were approved by the Institutional Animal Care and Use Committee of Shanghai Jiao Tong University.

### Cell lines and culture

GBC-SD and SGC996 cells were purchased from the Shanghai Cell Institute Country Cell Bank. The GBC-SD cells were cultured in high-glucose DMEM (Gibco, USA) and the SGC996 cells were cultured in RPMI1640 (Gibco, USA). All of the two cells were supplemented with 10% fetal bovine serum (Gibco, USA), 100 μg/mL streptomycin and 100 U/mL penicillin (Hyclone, USA), at 37°C, under a 5.0% CO2 atmosphere.

### Drugs and antibodies

**Figure 1:**
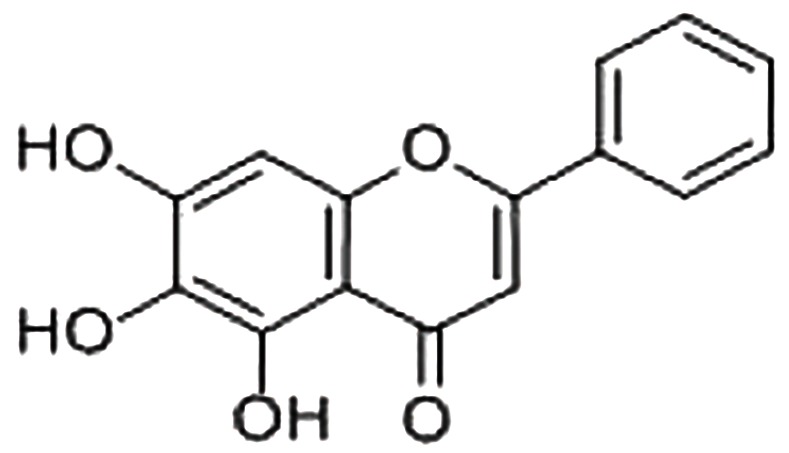
Chemical structure of baicalein. The molecular formula of baicalein is C15H10O5and its molecular weight is 270.24.

The baicalein was purchased from Sigma-Aldrich (St. Louis, USA),dissolved in DMSO as a stock concentration of 100 mmol/L, and stored in the dark at −20°C. Fig. 1 shows the chemical structural of baicalein. The final baicalein concentrations used for the different experiments were prepared by diluting the stock solution with high-glucose DMEM or RPMI1640. The antibodies used for western blotting were as follows: rabbit anti-caspase-3, anti-Bcl-2, anti-Bax, anti-PARP, anti-p53, anti-cyclinA, anti-cyclinB1, anti-cyclinD1, anti-MMP2, anti-MMP9, anti-ZFX and mouse anti-β-actin. All the antibodies were purchased from Cell Signaling Technology.

### 3-(4, 5-dimethylthiazol-2-yl)-2, 5 diphenyl tetrazolium (MTT) assay

Drug sensitivity was determined using the MTT assay. Briefly, cells were trypsinized and plated into 96-well plates (Corning, USA) at a density of 5 × 10^3^ cells per well. The cells were cultured overnight and then replenished with fresh medium containing various concentrations (0, 15, 30, 60, 120, and 240 μmol/L) of baicalein for 24, 48, and 72 h. Thereafter, 20μl of MTT (Sigma-Aldrich) dissolved in PBS at 5 mg/ml was added directly to all the wells, and the plates were incubated for 4 h at 37°C. The formazan crystals that formed were dissolved in 100 μl of DMSO after removal of the supernatant. The optical density was recorded at 490 nm on a microplate reader (Bio-Tek, USA). The results represent the average of 3 independent experiments done over multiple days. The percentage of cell viability was calculated as follows:

$$\text{cell viability}\;(\%)=\frac{\text{OD of treatment}}{\text{OD of control}}\times 100$$

### Colony forming assay

Cells in the logarithmic growth phase were digested into a single-cell suspension with a trypsin-EDTA (Gibco, USA) solution, and then 2 ml of the cell suspension was seeded onto 6-well plates (Corning, USA) at a density of 200 cells/ml. After adherence, the cells were treated with baicalein (0,6,12,and 24μmol/L) for 48 h and then cultured for 15days. Thereafter, the cells were fixed with 10% formalin and stained with 0.1% crystal violet (Sigma-Aldrich). After washing, the plates were air dried, and digital images were taken of stained single clones observed under a microscope (Leica, Germany). The results represent the average of 3 independent experiments done over multiple days.

### Cell cycle analysis

Cells were treated with baicalein (0,30,60 and 120μmol/L) for 48 h. Then, the cells were collected and fixed with cold 70% ethanol and stored at −20°C. The cells were then washed and resuspended in cold PBS and incubated at 37°C for 30 min with 10 mg/ml RNase and 1 mg/ml propidium iodide (Sigma-Aldrich). DNA content analysis was performed by flow cytometry (BD, San Diego, USA). The percentage of cells in the different cell cycle phases was determined using the Cell Quest acquisition software (BD Biosciences).

### Flow cytometry analysis of cell apoptosis

The annexin V/propidium iodide assay was performed according to the manufacturer’s recommendation (Invitrogen, USA). Briefly, cells were plated into 6-well plates (Corning, USA) and incubated for 48 h with baicalein (0,30,60,and 120μmol/l). In brief, the cells were collected and then were washed with cold PBS, centrifuged, resuspended in 100 ul of binding buffer containing 2.5ul FITCconjugatedannexin-V and 1ul 100 ug/ml PI and incubated for 15 mins at room temperature in the dark. A total of at least 10 000 events were collected and analyzed by flow cytometry (BD, San Diego, USA).

### Detection of morphological apoptosis with Hoechst 33342 staining

After treatment with baicalein (0,30,60,and 120μmol/L) for 48 h, the GBC-SD cells were washed with PBS and fixed with methanol:acetic acid (3:1) for 15 min at room temperature. Fixed cells were washed with PBS and stained with 5 μg/ml of Hoechst 33342 stain for 10 min. Changes in the nuclei of cells after staining with Hoechst 33342 were observed using a fluorescence microscope (Leica, Germany).

### Cell migration and invasion

GBC-SD3×10^4^ cells and SGC996 1×10^5^ cells were placed on the non-coated membrane in the top chamber (Corning, USA). Cells were plated in medium without serum. Medium containing 10% FBS was placed in the lower chamber to act as a chemoattractant. After adherence, cells were treated with various concentrations of Baicalein (0, 7.5, 15, 30, 60 μmol/L) in serum-free medium. The cells were incubated for 24 hours; cells that did not invade through the pores were removed using a cotton swab. Cells migrating through 8.0 μm polycarbonate membranes to the lower surface were fixed with 10% methanol and stained with 0.1% crystal violet. Migrating cells were quantified by counting stained cells under a microscope (× 200) in five random fields for each well. Each experiment was performed in triplicate. Invasion assays were performed in a similar manner to the migration assays, except that inserts were precoated with Matrigel (BD, USA).

### In vitro wound healing assay

GBC-SD cells were grown in 6-well plates at a density of 1.5 × 10^5^/mL and grown to confluence. Wound was created by scraping confluent cell monolayers with a pipette tip. Cells were extensively rinsed with medium to remove cellular debris before treating with different concentrations(0, 7.5, 15, and 30μmol/L) of baicalein in FBS deprived condition. The cells were allowed to migrate for 24 h and images of the migrated cells were taken using the inverted microscope (Leica, Germany).

### Reverse transcription and quantitative real-time polymerase chain reaction (qPCR)

Quantitative PCR was used to quantify the expression of mRNA in the experimental groups. Briefly, GBC cells were treated with baicalein (15, 30, 60 and 120 μmol/L) for 48 h. Total RNA was isolated using the RNA easy kit (Invitrogen, USA). First strand cDNA was synthesized from 500ng total RNA using a PrimeScript Reverse Transcriptase (TaKaRa, Japan). Quantitative real-time PCR was performed in a reaction volume of 20 μl including 2 μl cDNA. The primer sequences were as follows: MMP9 (forward-5’-TGT ACC GCT ATG GTT ACA CTC G-3’, reverse-5’-GGC AGG GAC AGT TGC TTC T-3’), MMP2 (forward-5’-AAC TAC GAT GAT GAC CGC AAG’;reverse-5’-GAC AGA CGG AAG TTC TTG GTG -3’),ZFX (forward-5’-TGT GGA GTG TGG TAA GGG TTT T’;reverse-5’-ACT GGT GTG TTT TGC TTT CTT G -3’), and GAPDH(forward-5’-AAG CTC ATT TCC TGG TAT GACA-3’, reverse-5’-TCT TAC TCC TTG GAG GCC ATGT-3’). PCR conditions were as follows: 95°C for 30sec followed by 40 cycles at 95°C for 5 sec, 60°C for 34 sec. Glyceraldehyde-3-phosphate dehydrogenase (GAPDH) was used as an internal reference gene to normalize the expression of apoptotic genes. Relative quantification of apoptosis-related genes was analyzed by the comparative threshold cycle (Ct) method. For each sample, the Ct value of the apoptotic gene was normalized using the formula: ΔCt = Ct (apoptotic genes) − Ct (GAPDH). To determine relative expression levels, the following formula was used: ΔΔCt = ΔCt (treated) − ΔCt (control). The value was used to plot the expression of apoptotic genes using the formula 2–ΔΔCt.

### Western blot analysis

Cells were treated with various concentrations of baicalein (0, 15, 30, 60 and 120 μmol/l) for 48 h and then lysed in a sample buffer, followed by denaturation. The total protein concentration of the cell extracts was determined using the bicinchoninic acid assay system (Beyotime, China) with BSA as a standard. Equal quantities (80 μg protein per lane) of total proteins were separated by SDS-PAGE (8%, 12% gels) under reducing conditions. The proteins were then electrophoretically transferred to nitrocellulose membranes. The membranes were blocked with 5% skimmed milk, and incubated withanti-caspase-3, anti-Bcl-2, anti-Bax, anti-PARP, anti-p53, anti-cyclinA, anti-cyclinB1, anti-cyclinD1, anti-MMP2, anti-MMP9, anti-ZFX antibodies, respectively (1:1000; Cell Signaling Technology) at 4°C overnight. This was followed by an incubation with goat anti-rabbit/anti-mouse secondary antibody conjugated with horseradish peroxidase (1:5000; Abcam). An equal loading of each lane was evaluated by immunoblotting the same membranes with β-actin antibodies after the detachment of previous primary antibodies. Photographs were taken and the optical densities of the bands were scanned and quantified with the Gel Doc 2000 (BioRad, USA).

### In vivo tumor xenograft study

Six-week-old male athymic nude mice (with an initial body weight of 20–22 g) were obtained from Shanghai SLAC Laboratory Animal Co., Ltd. (Shanghai, China) and housed under pathogen-free conditions with controlled temperature (22°C), humidity, and a 12 hour light/dark cycle. Food and water were given ad libitum throughout the experiment. SGC996 cells were used for tumor xenografts and grown in culture and then detached by trypsinization, washed, and resuspended in serum-free RPMI1640. Each of the athymic nude mouse was subcutaneously injected of 1 × 10^6^ of cells ina volume of 100ul in the right flank area to initiate tumor growth. After SGC996 inoculation, mice were randomly assigned to two groups (5 mice/group). One group was treated with vehicle (10% DMSO and 90% propylene glycol) intraperitoneally, and the other group received0.4mg/mouse baicalein intraperitoneally for 9 times. At the 21th day, the animals were sacrificed, and the tumor tissue was removed and weighed.

### Statistical analysis

All values are expressed as the mean ± SD and they were analyzed by the Student’s *t*-test using SPSS version 13.0 software. A *p*-value of less than 0.05 was considered significant.

## Results

### Effect of baicalein on the viability and apoptosis of gallbladder carcinoma cells in vitro and in vivo

**Figure 2:**
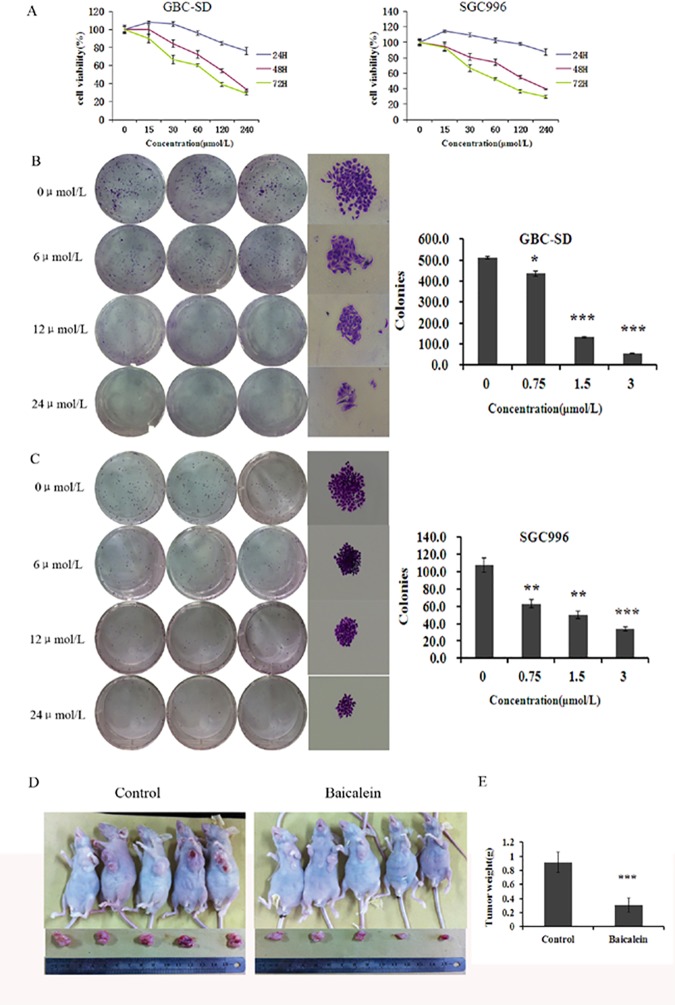
Baicalein inhibits the proliferation ofgallbladder carcinoma cells *in vitro* and *in vivo*. (A) GBC-SD and SGC996 cells were treated with varying concentrations of baicalein, and the cell proliferation were determined by MTT assay on days 1, 2, and 3. Each value represents the mean ± SD (n = 3). (B and C) Baicalein suppressed colony formation of GBC-SD and SGC996 cells. Cells were treated with baicalein (6, 12, and 24 μmol/L) and were allowed to form colonies in fresh medium for 14 days. The influence of colonies (mean ± SD, n = 3) in colony formation are shown. (D) Baicalein-treated xenograft tumors were much smaller than control tumors. (E) The weight of tumors revealed that xenograft tumor growth in nude mice was significantly slower in baicalein-treated nude tumors than control tumors. The results shown are representative of at least three independent experiments. *P < 0.05, **P < 0.01, ***P < 0.001.

The effects of baicalein on the growth of human GBC-SD and SGC996, two most widely used cell lines of gallbladder cancer, *in vitro* were tested. As shown in Fig. 2(A), after treatment for 24, 48, and 72 h, baicalein induced a dose- and a time-dependent decrease in the viability of both the GBC-SD and SGC996 cells, as analyzed by the MTT assay. As shown in Fig. 2(B)&(C), the ability of GBC-SD and SGC996 cells to form colonies in the presence of baicalein was detected with the flat plate colony formation assay. The colony count indicated that baicalein had induced a dose-dependent decrease in the colony formation ability. The findings support the fact that baicalein may exert a significant influence on GBC-SD and SGC996 cells proliferation. To further confirm the effect of baicalein *in vivo*, we tested the tumorigenicity of gallbladder carcinoma cells using nude mice tumor xenograft study. As shown in Fig. 2(D), the tumor size in group of baicalein treatment was smaller than that of the control group.

**Figure 3:**
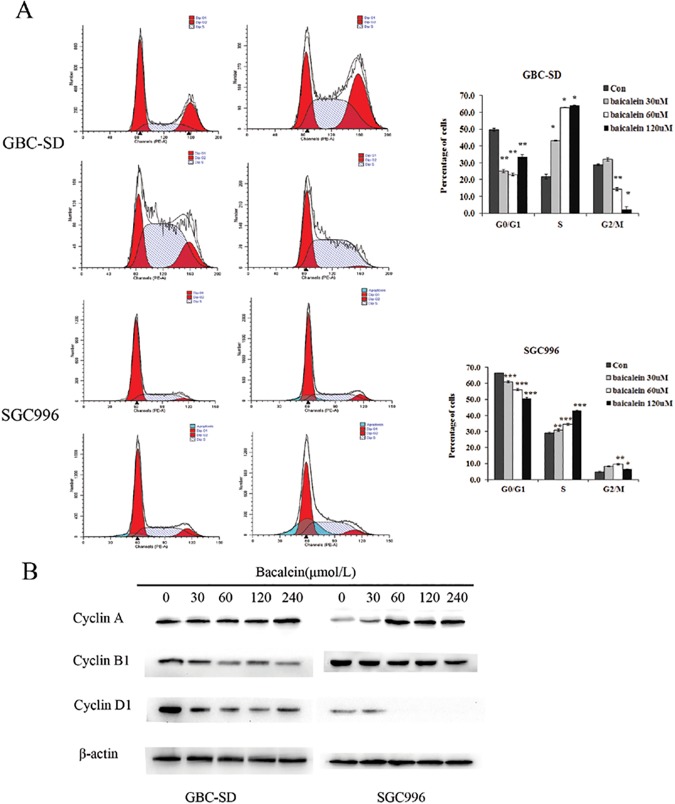
Baicalein induces S-phase arrest in GBC-SD and SGC996 cells. (A)Cells were treated with 30, 60, or 120 μmol/L baicalein for 48 h and the DNA content was analyzed by flow cytometry. The percentage of cells in the G1, S, and G2/M phases of the cell cycle are shown. These results were from 1 representative experiment of 3 independent trials. (B) Expression of cyclin B and cyclin D in GBC-SD cells and cyclin A and cyclin D in SGC996 cells were significantly changed after treatment with baicalein compared with control. The results shown are representative of at least three independent experiments. *P < 0.05, **P < 0.01, ***P < 0.001.

To assess whether baicalein affects cell cycle progression, flow cytometric analysis was carried out. The results showed a significant decrease in the number of cells in the proliferative G0/G1 phase and a significant increase in the number of cells in the S phase, after 48 h of treatment with baicalein (Fig. 3(A)). CyclinA, a G1/S transition promotion protein, was dose-dependently increased by baicalein treatment. It may be the reason of S phase arrest induced by baicalein. CyclinB1 and cyclinD1 which represent S/G2 and M/G1 transition process respectively, were dose-dependently decreased by baicalein treatment (Fig. 3(B) and S5 Fig.). These results indicate that baicalein arrests the cell cycle at the S phase.

**Figure 4:**
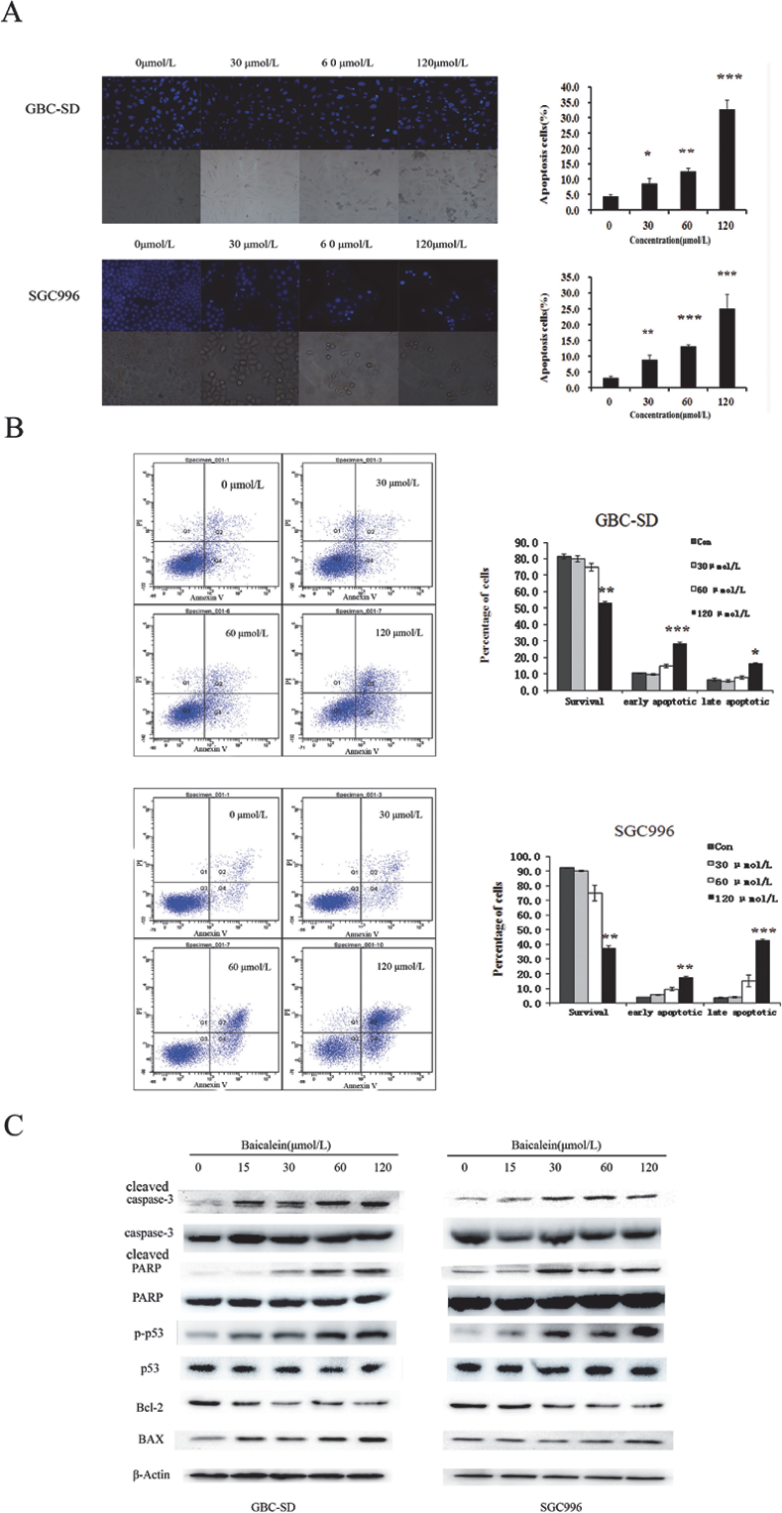
Baicalein induces apoptosis in GBC-SD and SGC996 cells. (A) Apoptotic morphological changes such as abnormal nuclear morphology, reduction in cell number with apoptotic body formation, and cell shrinkage, induced by baicalein (30,60 and 120 μmol/L) treatment for 48 h, were observed by Hoechst 33342 staining in GBC-SD and SGC996 cell lines. (B) Cells were incubated with baicalein (30,60 and 120 μmol/L) for 48 h, followed by staining with annexin-V/PI. Cell apoptosis was estimated by the flow cytometry. (C) Baicalein induces the activation of apoptosis-related proteins in GBC-SD and SGC996 cells. After treatment with baicalein (0, 15, 30, 60, and 120μmol/L) for 48 h, cell lysates were prepared and western blot analysis was performed against Bcl-2, Bax, pro-caspase-3, P53 and PARP. β-Actin was used as a loading control. The results shown are representative of at least three independent experiments. *P < 0.05, **P < 0.01, ***P < 0.001.

To further confirm these results, we evaluated the effects of baicalein on apoptosis in GBC-SD and SGC996 cells by using annexin V-FITC and propidium iodide staining. As assessed by flow cytometry and shown in Fig. 4(B), a marked dose-dependent increase in both the early and late stages of apoptosis was obvious in GBC-SD and SGC996 cells after baicalein treatment compared with control cells.

Morphological changes in the apoptotic cells were revealed by the Hoechst 33342 staining, as shown in Fig. 4(A). In the untreated GBC-SD and SGC996 cells, the nuclei were stained weak homogeneous blue, whereas in the group treated with baicalein, bright chromatin condensation and nuclear fragmentation were observed.

### Effect of baicalein on the apoptosis related factors

To identify which proteins were mainly involved in the baicalein-induced apoptotic process, we analyzed a number of apoptosis related factors by western blot. As illustrated in Fig. 4(C), pro-caspase-3 and P53 were down-regulated by baicalein treatment. Cleaved PARP was significantly increased. Protein level of Bax was up-regulated, while the protein level of Bcl-2 was significantly decreased.

### Effect of baicalein on the metastasis of gallbladder carcinoma cells

**Figure 5:**
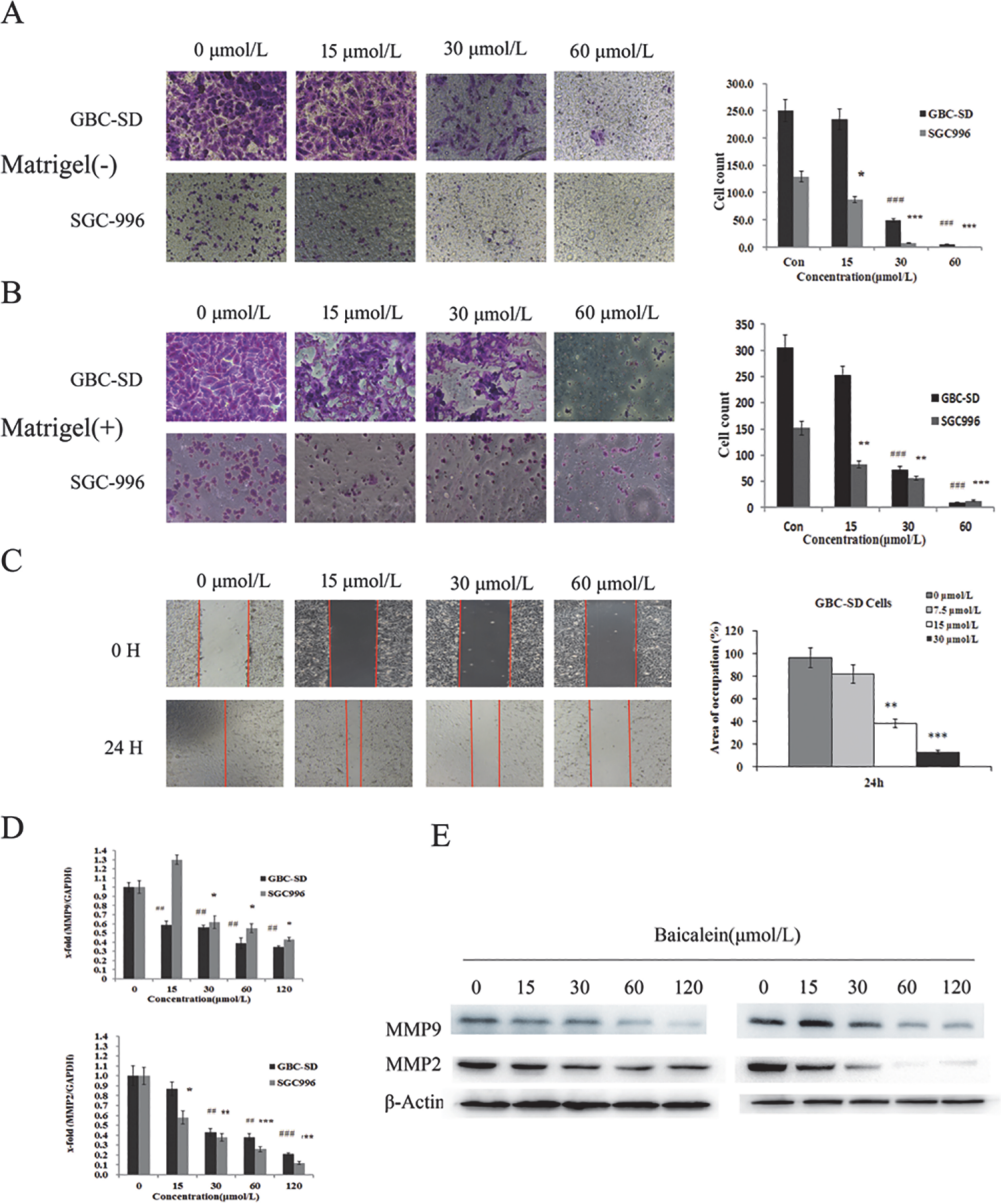
Effect of baicalein on the mobility and invasive potency of human gallbladder carcinoma GBC-SD and SGC996 cells. Cells were treated with 7.5, 15, 30 and 60μmol/L baicalein for 12 h prior to the migration (A) or invasion (B) assay.(C) Cell monolayers were wounded by scratching with a 200 μl pipette tip. The occupation area in GBC-SD cell monolayer treated with baicalein was dose dependent decreased 24 h after scratch. Baicalein affects the expression of matrix metallopeptidases (MMPs). (D) The mRNA expression of MMP-2 and MMP-9 was detected by qPCR after treated with baicalein for 48h. (E) The protein expression of MMP-2 and MMP-9 was detected by western blot after treated with baicalein for 48h. The results shown are representative of at least three independent experiments. *P < 0.05, **P < 0.01, ***P < 0.001.

Since gallbladder carcinoma cell migration and invasion are associated with metastasis, we used various cellular assays to evaluate the effects of baicalein on the metastatic potential of gallbladder carcinoma cells. To examine the effect of baicalein on cancer cell mobility, we performed migration assay using GBC-SD and SGC996 cells. As shown in Fig. 5(A), cellular migration was inhibited by baicalein treatment. Moreover we performed wound healing assay to further confirm the inhibition effect of baicalein on cell migration. As shown in Fig. 5(C), baicalein conspicuously inhibited the GBC-SD cell migration in a dose-dependent manner. To further explore whether the invasive activity was affected in response to baicalein, we examined the invasive activity using a matrigel coated membrane. The results showed that baicalein dramatically reduced the number of invaded cells and this inhibition occurred in a concentration-dependent manner (Fig. 5(B)). Furthermore, the proliferation and metastasis abilities were further potentiated in cells treated with both siRNA against ZFX and baicalein (S4 Fig.).

MMPs are known to be crucial for degrading extracellular matrix components and for promoting tumor cellular invasion *in vitro* and *in vivo*. After treatment with different concentration of baicalein for 48 h in GBC-SD and SGC996 cells, quantitative real-time PCR and western blot analysis showed that the mRNA and protein expression of MMP-9 and MMP-2 significantly decreased compared to the control group in a dose-dependent manner (Fig. 5(D&E)).

### Baicalein down-regulated zinc finger X-chromosomal(ZFX) protein and mRNA expression in GBC-SD and SGC996 cells

**Figure 6:**
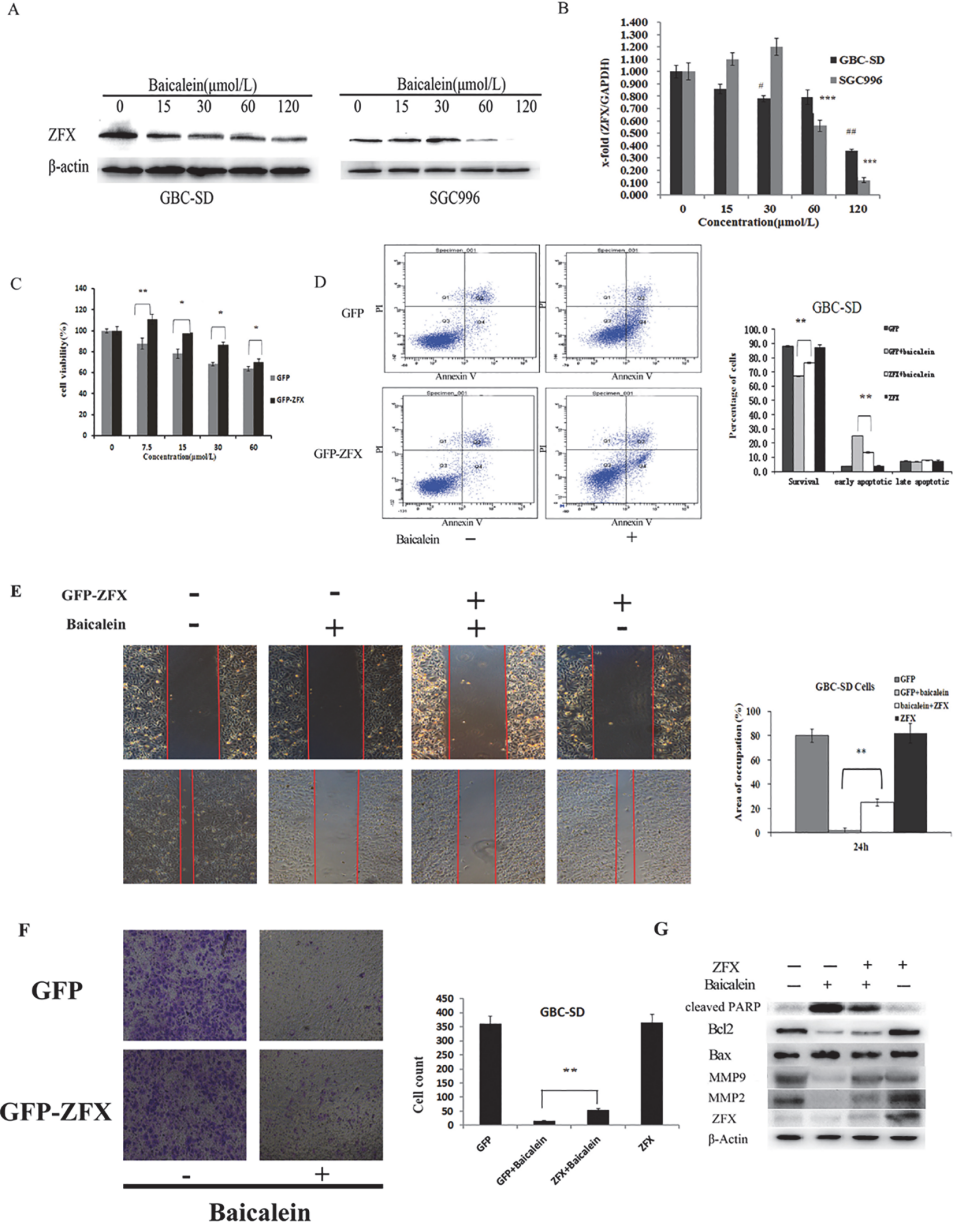
Effect of ZFX on the proliferation, apoptosis, invasive activity and related protein signaling of baicalein-treated GBC-SD cells. (A and B) ZFX expression in GBC-SD and SGC996 cells induced by baicalein. Cells were treated with 0, 15, 30, 60 and 120 μmol/L baicalein for 48h, then the protein and mRNA expression level of ZFX was determined by Western blot(A) and qPCR(B). (C and D) Cells were transfected with ZFX-GFP plasmid and GFP plasmid, respectively. 72 hours after transfection, cells were treated with 0, 7.5, 15, 30 and 60 μmol/L baicalein for 72h. ZFX-GFP significantly blocked the inhibition of proliferation of GBC-SD cells by baicalein using MTT assay (C). ZFX-GFP significantly blocked the induction of apoptosis of GBC-SD cells by baicalein estimated by the flow cytometry(D). (E and F) 72 hours after transfection, cells were treated with 60 μmol/L baicalein for 24h. ZFX-GFP significantly blocked the anti-migration and invasion effect of GBC-SD cells by baicalein. The cell migration was estimated by wound healing assay(E) and the cell invasion was estimated by transwell assay(F). (G) ZFX-GFP prevented the expression of PARP, the inhibition of MMP2, MMP9 and up-regulated the ratio of Bcl2 /Bax in response to baicalein. 72 hours after transfection, cells were treated with 60 μmol/L baicalein for 48h.The protein levels of PARP, MMP-2, MMP-9,Bcl2, Bax and ZFX were determined by Western blot. The results shown are representative of at least three independent experiments. *P < 0.05, **P < 0.01, ***P < 0.001.

Our previous study found that knock-down of ZFX could inhibit GBC-SD cell proliferation and migration. So we are curious about whether the effect of baicalein on gallbladder carcinoma cells is connected to ZFX expression. Western blot and quantitative real-time PCR analysis revealed that the protein and mRNA expression of ZFX was dose-dependently (Fig. 6(A&B)) decreased by baicalein treatment in GBC-SD and SGC996 cells and the translocation of ZFX into nuclei was suppressed by baicalein treatment (S2 Fig.). The effect of baicalein on ZFX expression was specific because the expression level of NF-κB and STAT3 were not affected by this molecule (S1 Fig.).

### Overexpression of ZFX relieved the viability, apoptosis and metastasis effect of baicalein in GBC-SD cells

Furthermore, overexpression of ZFX (S3 Fig.) resulted in partially blocking of baicalein induced proliferation and apoptosis in GBC-SD cells (Fig. 6(C)&(D)). Consequently, the inhibition of migration (Fig. 6E) and invasion (Fig. 6F) abilities of cells by baicalein were remarkably reversed by the overexpression of ZFX. On the other hand, overexpression of ZFX greatly inhibited baicalein induced activation of PARP and increased the ratio of Bcl2/Bax. Furthermore, the overexpression of ZFX also resulted in blocking of baicalein induced inhibition of MMP-2 and MMP-9(Fig. 6(G)). Thus, the data suggested that the ZFX protein might participate in baicalein-induced cell apoptosis and anti-metastasis in gallbladder carcinoma cells.

## Discussion

Our study provides some new information about baicalein used in the anti-cancer therapy. The anti-cancer effect of baicalein has been confirmed in many other cancers, but the anti-proliferation and metastatic effect and the related mechanism(s) in GBC cells is not clear. Here, we have shown the biochemical and molecular mechanisms of apoptosis and anti-metastatic induction by baicalein.

Firstly, we found that baicalein may play an important role in GBC cell proliferation, cell cycle and apoptosis *in vitro* and *in vivo*. Treatment with baicalein resulted in a marked decrease in the viability of cultured GBC-SD and SGC996 cells and in the number of colonies that these cells formed. To our knowledge, this is the first study to evaluate the potential role of baicalein on *in vivo* xenograft animal model. Significant reduction of tumor mass was observed after a 3-week treatment. The *in vivo* effect of baicalein on GBC tumors strongly support baicalein as a potential new drug for anti-GBC treatment.

The effect of baicalein on cell cycle arrest and the induction of apoptosis in GBC cells was also evaluated. The anti-tumor effects of baicalein were believed to be used by influencing arrest in the cell cycle or by interacting with cell cycle-related proteins[31]. In our study, baicalein also induced S phase cell cycle arrest and inhibition of cyclin B1, cyclin D1, promotion of cyclin A in GBC-SD and SGC996 cells. Since apoptosis is considered as an important mechanism in the inhibition of cancer, we performed hoechst33342 staining assay, annexin V/PI assay, and detection of apoptosis related protein expression to further explore the mechanism of baicalein-induced apoptosis. Baicalein remarkably induced the apoptosis of both GBC-SD and SGC996 cells, as demonstrated by changes in nuclear morphology, an increase in the percentage of Annexin V-staining cells, which was further confirmed by enhanced expression of cleavage of pro-caspase-3, cleavage of PARP, Bax and reduced expression of Bcl-2 and P53. Many of these genes are related to the mitochondrial apoptotic pathway. Cleavage of pro-caspase-3, cleavage of PARP and ultimately degradation of DNA. Bax and Bcl-2 proteins, which regulate the essential change in mitochondrial membrane permeability for apoptosis[32]. From these data, it may be concluded that baicalein induced GBC cell apoptosis by activating the intrinsic mitochondrial pathway.

Besides cell proliferation, migration and invasive capacity of cells are also two of the most important features of malignant cell behavior. To elucidate the effect of baicalein on cell motility, cell migration and invasiveness were determined by a wound healing migration assay and transwell assay, respectively. From these data, we found that baicalein could suppress the migration and invasion capability of GBC-SD and SGC996 cells. MMPs are a highly regulated superfamily of zinc dependent endopeptidases that are causally associated with the development and progression of tumors[33]. It has been demonstrated that MMP-2 and MMP-9 expression is positively correlated with the depth of invasion, lymph node metastasis, and vessel permeation[34, 35]. In the present data, we found that baicalein could reduce the protein expression and enzymatic activity of MMP2 and MMP9. Taken together, our results suggested that baicalein plays an important role in inhibition of GBC cell migration and invasion.

To further dissect the molecular mechanisms underlying the anti-proliferation and invasive activity of baicalein, we focused on the role of ZFX, a zinc finger protein. Firstly, we found that both ZFX mRNA and protein were remarkably down-regulated by baicalein. Secondly, overexpression of ZFX remarkably decreased the number of apoptotic cells induced by baicalein treatment, which was further confirmed by reduced expression of Bax and enhanced expression of Bcl-2. Finally, overexpression of ZFX significantly weakened the inhibitive effect of metastatic activity induced by baicalein *in vitro*. ZFX was over-expressed in various tumor tissues compared to healthy tissues and had important effect on tumor proliferation and metastasis[28]. Thus, ZFX may be responsible for the anti-proliferation and invasive activity of baicalein.

Taken together, our results contribute that the capacity of baicalein to inhibit cell proliferation and metastasis in GBC cells is correlated with its ability to down-regulate ZFX.

## Supporting Information

S1 Fig The expression of STAT3 and NF-κB were not affected after the incubation with different concentration of Baicalein. Gallbladder cancer cells were treated with 15, 30 or 60 μmol/L baicalein for 48h and the expression level of STAT3 and NF-κB were analyzed by Western Blot.
(TIF)

S2 Fig Baicalein treatment suppressed the translocation of ZFX into nuclei. The cytoplasmic fraction and nuclear fraction were separated by NE-PER Nuclear and Cytoplasmic Extraction Kit (Thermo-Pierce Company) and the expression level of ZFX was detected in these two fractions by Western Blot.
(TIF)

S3 Fig Expression level of ZFX in cells transfected with ZFX-GFP plasmid. ZFX overexpression level was detected in control cells, cells transfected with empty vector and cells transfected with ZFX-GFP plasmid.
(TIF)

S4 Fig Knockdown of ZFX by using siRNA can potentiate baicalein-induced cell proliferation and metastasis. A) The expression level of ZFX was detected in different conditions. B-D) The proliferation(B) and metastasis(C and D) abilities of cells with siRNA against ZFX and baicalein were significantly potentiated.
(TIF)

S5 Fig CyclinA was dose-dependently increased by baicalein treatment in GBC-SD cells.
(TIF)
