# Supplementary figures and images for: Baicalein Inhibits Progression of Gallbladder Cancer Cells by Downregulating ZFX
Source: PLoS One. 2015 Jan 24;10(1):e0114851. doi: 10.1371/journal.pone.0114851 (PMC4305301; doi:10.1371/journal.pone.0114851)

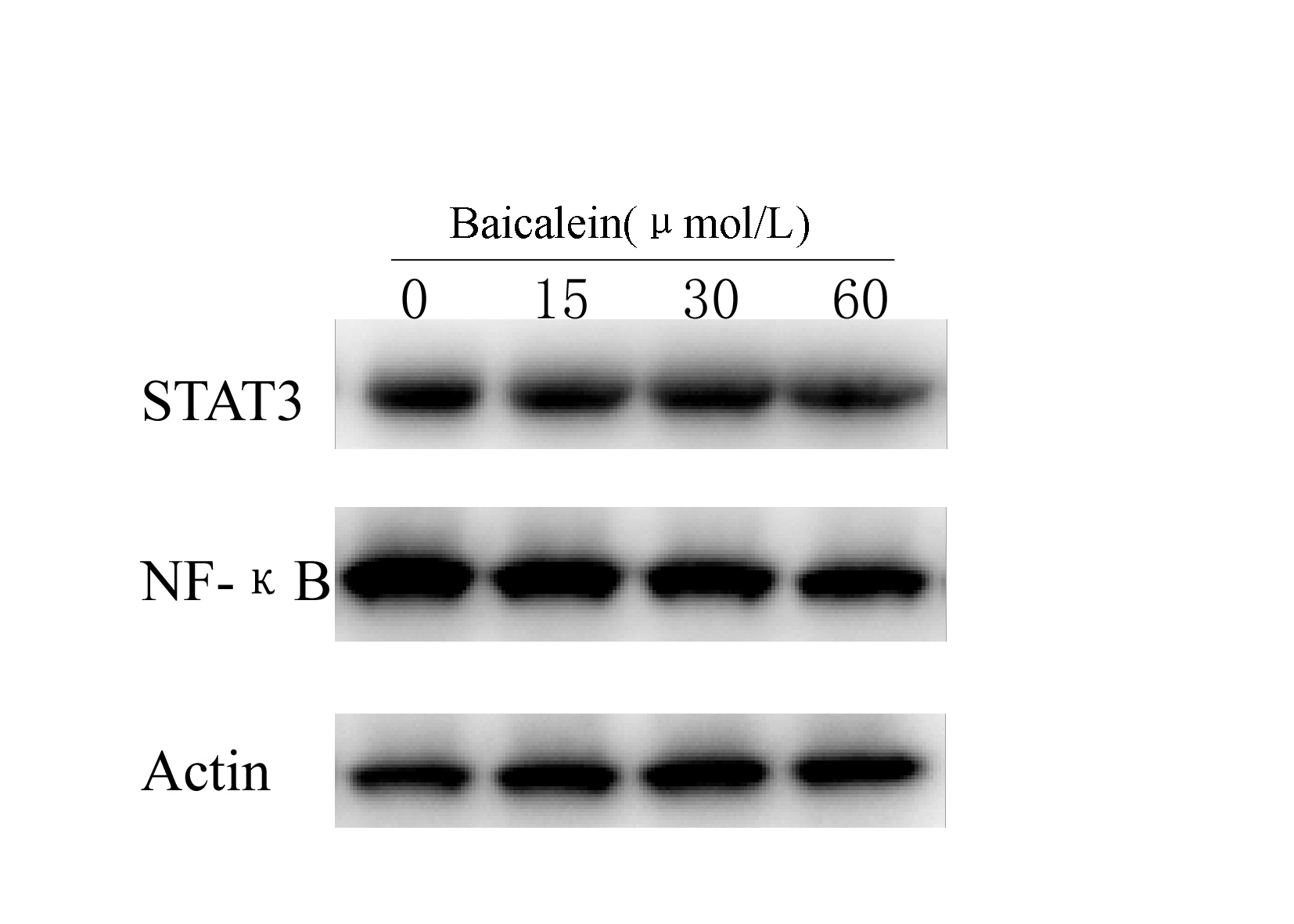

Supplement: S1 Fig — Gallbladder cancer cells were treated with 15, 30 or 60 μmol/L baicalein for 48h and the expression level of STAT3 and NF-κB were analyzed by Western Blot. (TIF) [file pone.0114851.s001.tif]

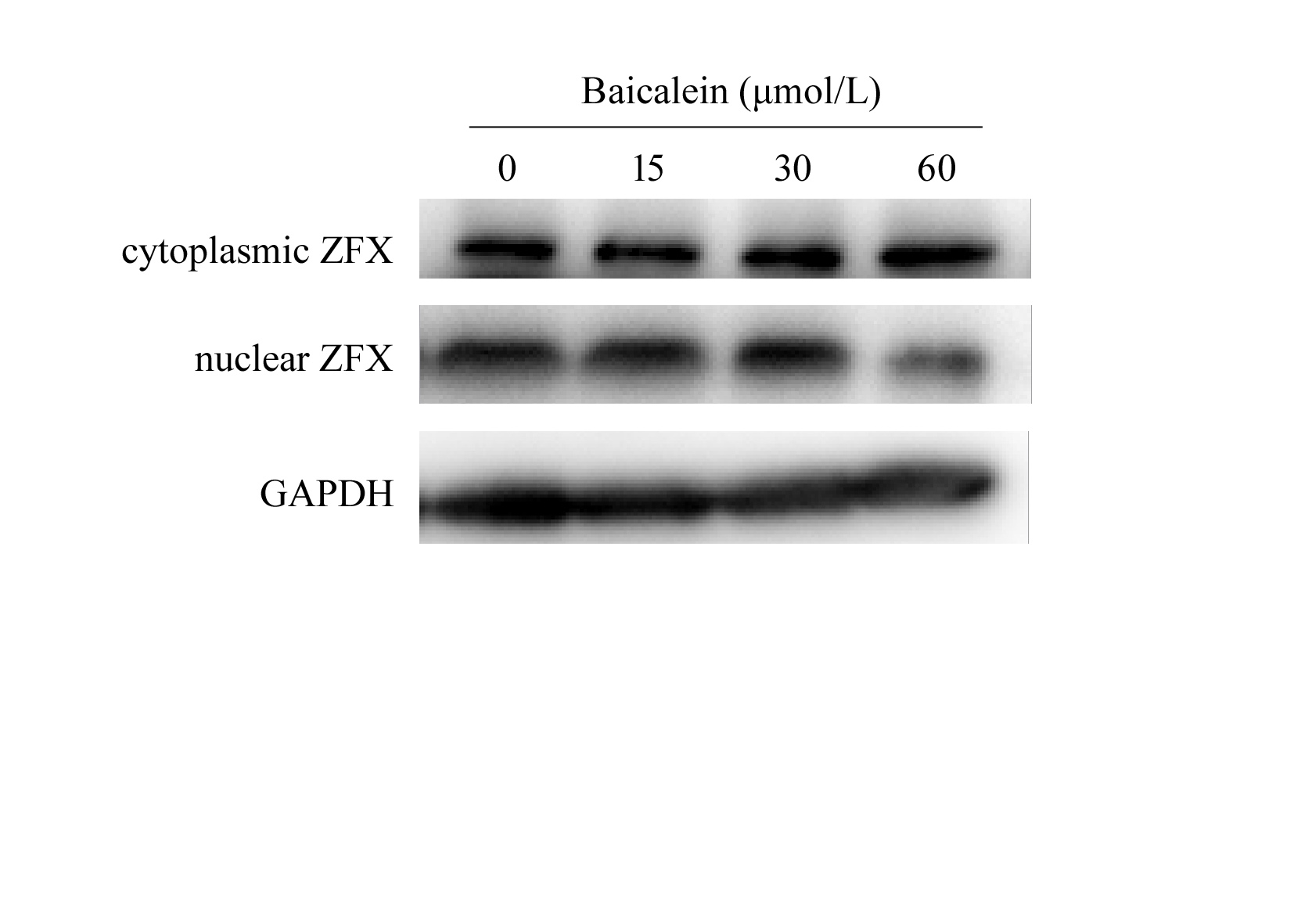

Supplement: S2 Fig — The cytoplasmic fraction and nuclear fraction were separated by NE-PER Nuclear and Cytoplasmic Extraction Kit (Thermo-Pierce Company) and the expression level of ZFX was detected in these two fractions by Western Blot. (TIF) [file pone.0114851.s002.tif]

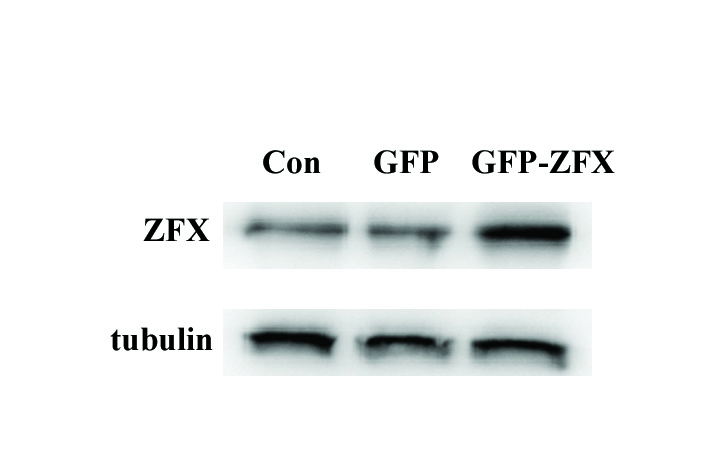

Supplement: S3 Fig — ZFX overexpression level was detected in control cells, cells transfected with empty vector and cells transfected with ZFX-GFP plasmid. (TIF) [file pone.0114851.s003.tif]

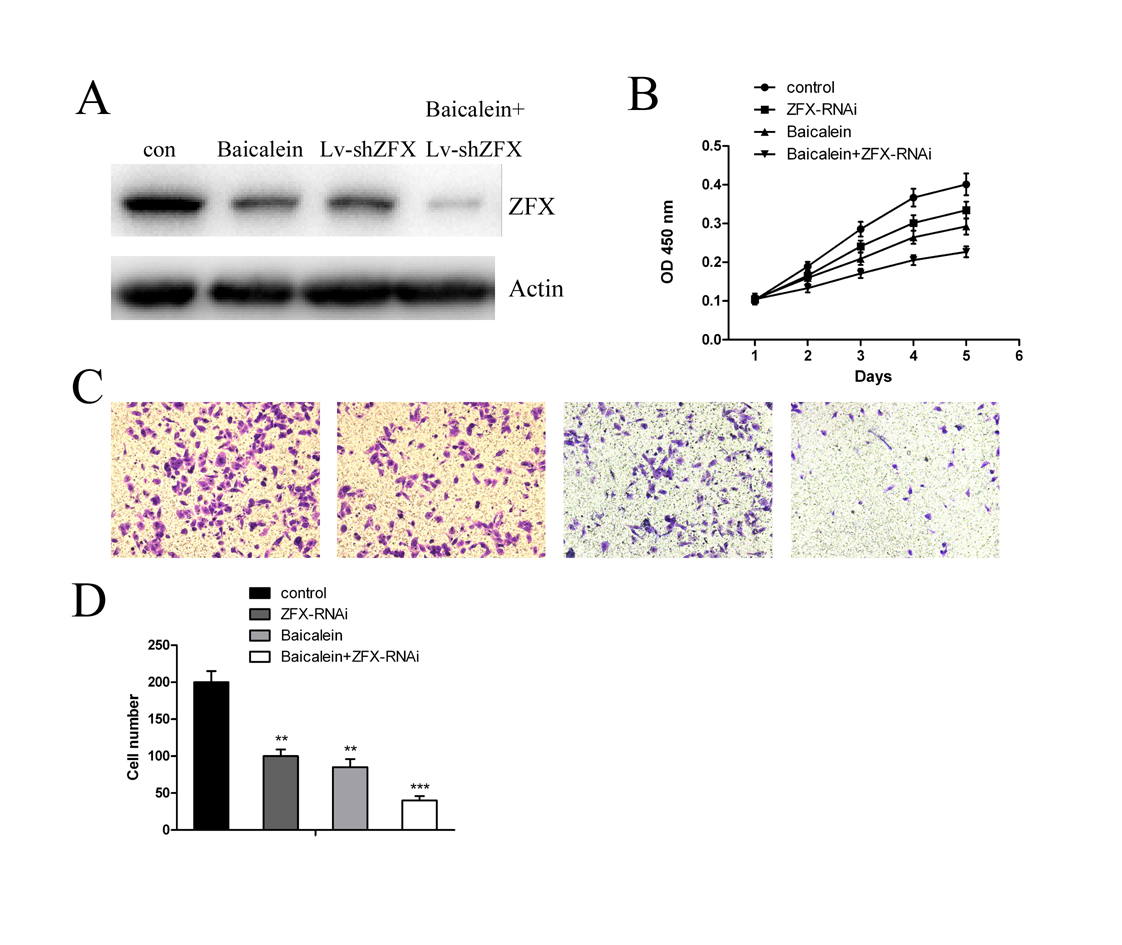

Supplement: S4 Fig — A) The expression level of ZFX was detected in different conditions. B-D) The proliferation(B) and metastasis(C and D) abilities of cells with siRNA against ZFX and baicalein were significantly potentiated. (TIF) [file pone.0114851.s004.tif]

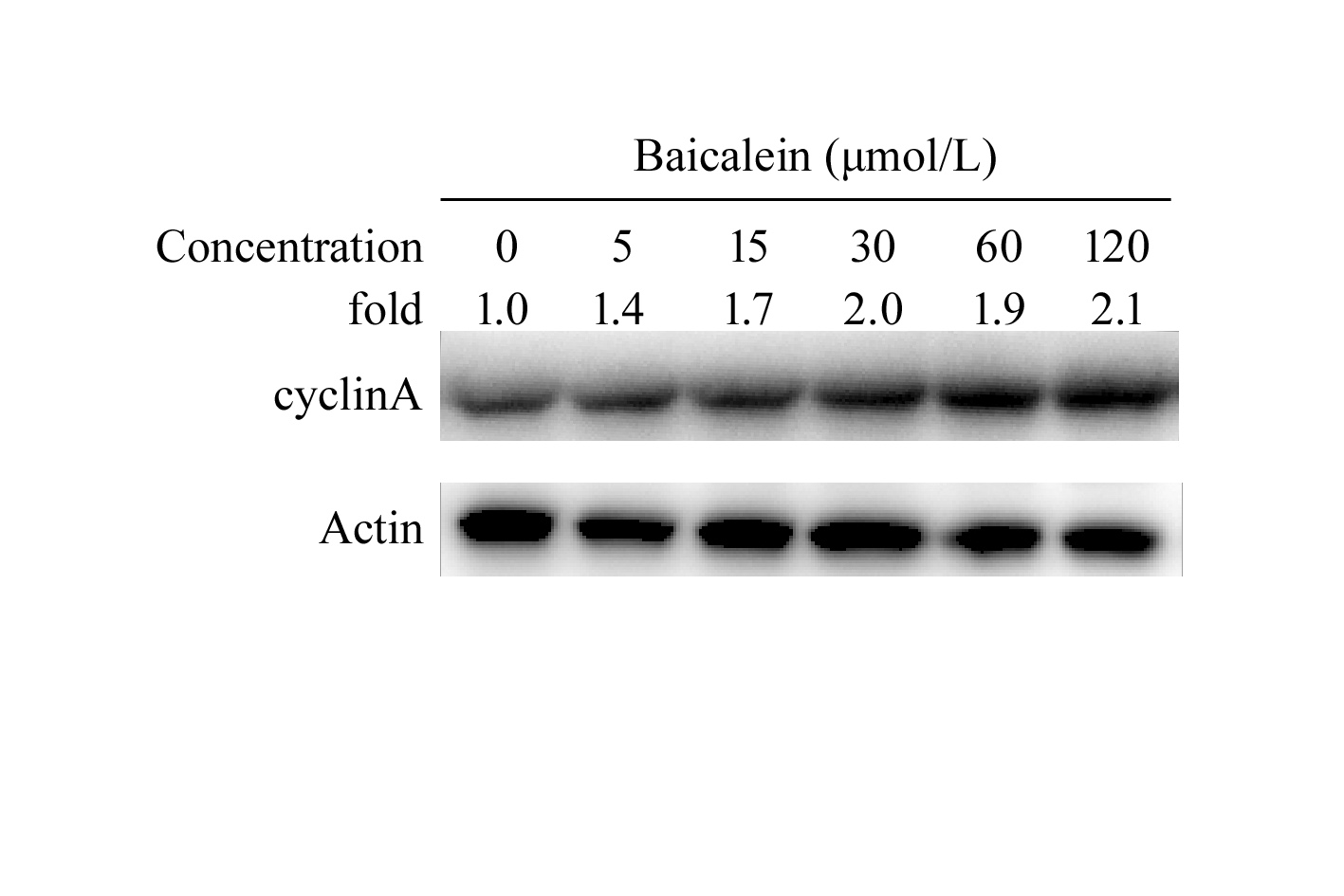

Supplement: S5 Fig — CyclinA was dose-dependently increased by baicalein treatment in GBC-SD cells. (TIF) [file pone.0114851.s005.tif]
